# A Journey to the Early Detection of Alzheimer's Disease in a Safety Net Hospital

**DOI:** 10.1002/alz70860_100457

**Published:** 2025-12-23

**Authors:** Sakina Ouedraogo Tall, Jillian Diuguid‐Gerber, Miesha Etheridge, Saima Ajmal

**Affiliations:** ^1^ New York University Grossman School of Medicine, New York, NY, USA; ^2^ New York City Health and Hospitals, New York, NY, USA

## Abstract

**Background:**

Nearly 7 million Americans are living with Alzheimer's Disease (AD) and it is a growing problem, particularly in racial minorities where disparities exist in the detection and diagnosis of AD. New York City Health and Hospitals (H+H) is the largest municipal health system in the nation and serves a diverse patient population. Over the last two years, H+H has joined the effort for the early detection of AD, creating an algorithm for the detection and management of Mild Cognitive Impairment and Dementia in primary care, implementing a telemedicine cognitive geriatric program, and receiving Age‐Friendly recognition at one of its largest facilities, Bellevue. We aim to expand this early detection initiative to H+H/ Woodhull as part of the Davos Alzheimer's Collaborative Healthcare System Preparedness (DAC‐SP) U.S. Early Detection Fellowship Program.

**Method:**

Using our experience and the DAC‐SP Early Detection Blueprint, we will implement the Annual Wellness Visit (AWV) at Woodhull Geriatrics Practice, then expand to those receiving care at the Adult Primary Care Practice. The AWV includes a brief cognitive screening tool (picture‐based memory impairment screening PMIS) and a functional assessment performed by the nursing staff and reviewed by the Primary Care Provider (PCP). Notes, templates, and smart phrases are built into the electronic medical system.

**Result:**

H+H aims to screen 15% of the diverse population currently receiving care at Woodhull Geriatrics Practice. This population consists of 27.7% Black, 58.5% Hispanic, 3.5 % non‐Hispanic White, and 1.7% Asian patients; 72% have Medicare, 12.4% Medicaid, and 12.1% are uninsured. We then plan to expand screening to a larger, but similarly diverse group of patients aged 65 and older receiving care in the Adult Primary Care Practice.

**Conclusion:**

H+H is dedicated to early detection of AD and related dementias. Building on the success of our program at a major facility, we are ready to tackle new challenges in expanding this vital initiative to the racially and ethnically diverse patient population at Woodhull and other locations.

